# Editorial: Translational neuroeconomic approach: from economic decision making to neuropsychological disorders

**DOI:** 10.3389/fneur.2025.1546863

**Published:** 2025-01-21

**Authors:** Mrinalini Srivastava, S. Senthil Kumaran, Achal Kumar Srivastava, Sanjay Singh

**Affiliations:** ^1^Department of Electrical Engineering, Indian Institute of Technology Delhi, New Delhi, India; ^2^Department of NMR, All India Institute of Medical Sciences, New Delhi, India; ^3^Department of Neurology, All India Institute of Medical Sciences, New Delhi, India; ^4^Department of Neurology, Creighton University, Omaha, NE, United States

**Keywords:** neuroeconomics, neuroimaging, decision making, game theory, psychopathological disorders, impulsive, reward processing

Neuroeconomics seeks to elucidate the neural mechanisms underlying human decision-making emphasizing on the cognitive processes that govern reward and risk-taking behavior. The integration of behavioral economics with neuroimaging techniques exhibits a comprehensive understanding of the complex interactions among morphological, anatomical and physiological states of brain leading toward a particular behavior. Thus, the interdisciplinary approach offers a transdiagnostic perspective on psychopathology by revealing shared underlying mechanisms of aberrant reward processing across multiple neurological and psychiatric conditions.

Neuroeconomics employs a reductionist method that combines economic, psychological, and neuroscientific levels of analysis to construct explanatory models of human choice behavior. The elucidation of neural mechanisms underlying decision-making has significant implications for the development of novel diagnostic and therapeutic approaches for psychopathology. Furthermore, neuroeconomics has the potential to reveal new insights into the biological underpinnings of psychiatric disorders ultimately contributing to the development of more effective treatments (1). Over the past decade, neuroeconomics research has predominantly concentrated on investigating decision-making processes in healthy population. However, a burgeoning interest in extrapolating neuroeconomic methodologies to psychiatric populations has the potential to explain the complex interplay between reward processing and decision-making in psychiatric disorders. Neuroeconomics relies on embedded psychopathological components for emotional processing and valuation. Economic utility framework offers an approach for understanding probabilistic sequential reinforcement in social context. Jorge et al. (2) used an economic trust game for a plausible explanation of decision-making under uncertainty in Diabetes Type 1. Furthermore, neural substrates of decision-making subsequent to Type 1 Diabetes Mellitus is conferred to limbic and reward related dopaminergic release (3). Therefore, endophenotypes of behavioral and neuroimaging origin shall provide a direct implication in addressing the impulsivity in Type 1 Diabetes Mellitus (4). Neuroeconomics approach can distinguish biobehavioral endophenotypes to explore the underlying neural mechanisms of decision-making under uncertain and ambiguous conditions (5). This special issue seeks to demonstrate the utility and efficacy of neuroeconomics approach in the neural mechanisms underlying reward-related decision-making in psychopathological disorders. It includes three original articles and one mini review contributing from neuroeconomics perspective for decision-making in Attention Deficit hyperactive disorder, delay discounting, temporal discounting, and burgeoning stroke cases due to high sodium intake and policymaking.

Neuroeconomics holds a considerable potential to be used as the translational approach by bridging the gap between economic decision-making and psychopathological disorders. However, several methodological constraints currently impede progress. The insufficiency of current research instruments for clinical translation is a significant drawback highlighting the need for more advanced and adaptable assessment tools that can be successfully used for diverse patient population. Neuroeconomics may overcome this constraint and progress the discipline by offering a thorough and integrative framework for studying decision-making processes. For instance, neuroimaging techniques (fMRI, EEG, etc.) are used in behavioral economic experiments to capture associated brain activity and parameterize components of reward-related decision-making (6). Therefore, application of neuroeconomic approaches to psychiatric disorders has the potential to provide promising candidate endophenotypes that may help to classify the basis of high heritability associated with psychiatric disorders. By examining psychopathology as a deviation from optimal behavior, neuroeconomics game theory approach can provide a more nuanced understanding of the complex interactions between brain behavior and decision-making (7).

Patients with psychopathological disorders such as substance abuse often exhibit impulsive decision-making under uncertain conditions. However, the extent to which these individuals are averse to ambiguous or uncertain situations still remains poorly understood. Recent research suggests that ambiguity is mediated by distinct neuropsychological processes that differ from those involved in decision-making under probabilistic uncertainty (8). The integration of economic decision theory with neuroscience has the potential to study and characterize important aspects of psychiatric disorders. Economic decision theory provides a framework for examining the complex interactions between social, psychological, and biological factors that underlie psychopathology. The current collection of the articles has contributed significantly in acknowledging neuroeconomics as a transdiagnostic approach for elucidating psychopathology of disproportionate reward processing into components such as subjective valuation, temporal discounting, delay discounting, hedonics, and reinforcement learning.

Chachar and Shaikh examines the importance of interdisciplinary research integrating neuroscience, psychology, and economics to develop effective interventions and support strategies for individuals with ADHD. Attention Deficit Hyperactivity Disorder (ADHD) significantly affects decision-making processes leading to substantial economic consequences. Neuroeconomic studies suggest that ADHD influences economic decision-making with particular components in impulsivity, risk-taking, and reward processing. Individuals with ADHD may exhibit suboptimal decision-making by selecting options that do not maximize expected value and may be related to specific brain regions prefrontal cortex, striatum, and anterior cingulate cortex that play significant role in executive functions. The article by Jung and Kim used graph theoretical analysis for comparing global and regional topological properties on impulsive choices between high discounting group (HDG) and low discounting group (LDG) individuals with high and low delay discounting tendencies. The results show that individuals with high delay discounting (HDG) indicate low network segregation and high integration with lower betweenness centrality in parahippocampal gyrus and amygdala. This study significantly highlights the role of underlying neural mechanisms driving the impulsive choice and delay discounting. Haj and Moustafa observed significant positive correlation in short term investors with higher temporal discounting for both money and Bitcoin. This further demonstrates that Bitcoin holders with short time horizons prioritize immediate gains over larger delayed gains.

The article by Zhang et al. examined the global, national, and regional impact of high-sodium diets (DHIS) on the burden of stroke. The Global Burden of Diseases Study 2019 data findings suggest that DHIS accounted for 17,673.33 million disability-adjusted life years (DALYs) and 700.98 thousand deaths from stroke in 2019. The study highlights the urgent need for effective interventions to alleviate the burden of stroke associated with DHIS, particularly in regions with moderate sociodemographic indexes (SDI) values and among males.

Ultimately, a decision-theoretic perspective for characterization of reward-specific stimuli that increase the risk of neurological and psychiatric disorders is needed to optimal decision-making. Neuroeconomics approach offers new avenues psychopathological studies for the development of novel diagnostics and interventions.
